# Chemistry-mediated Ostwald ripening in carbon-rich C/O systems at extreme conditions

**DOI:** 10.1038/s41467-022-29024-x

**Published:** 2022-03-17

**Authors:** Rebecca K. Lindsey, Nir Goldman, Laurence E. Fried, Sorin Bastea

**Affiliations:** 1 Physical and Life Sciences Directorate, Lawrence Livermore National Laboratory, Livermore, CA 94550 USA; 2 Department of Chemical Engineering, University of California, Davis, CA 95616 USA

**Keywords:** Density functional theory, Molecular dynamics

## Abstract

There is significant interest in establishing a capability for tailored synthesis of next-generation carbon-based nanomaterials due to their broad range of applications and high degree of tunability. High pressure (e.g., shockwave-driven) synthesis holds promise as an effective discovery method, but experimental challenges preclude elucidating the processes governing nanocarbon production from carbon-rich precursors that could otherwise guide efforts through the prohibitively expansive design space. Here we report findings from large scale atomistically-resolved simulations of carbon condensation from C/O mixtures subjected to extreme pressures and temperatures, made possible by machine-learned reactive interatomic potentials. We find that liquid nanocarbon formation follows classical growth kinetics driven by Ostwald ripening (i.e., growth of large clusters at the expense of shrinking small ones) and obeys dynamical scaling in a process mediated by carbon chemistry in the surrounding reactive fluid. The results provide direct insight into carbon condensation in a representative system and pave the way for its exploration in higher complexity organic materials. They also suggest that simulations using machine-learned interatomic potentials could eventually be employed as *in-silico* design tools for new nanomaterials.

## Abstract (web summary)

Modelling the growth of carbon nanoclusters in shock experiments is computationally demanding. Here the authors employ a machine-learned reactive interatomic model to perform large-scale simulations of nanocarbon formation from prototypical shocked C/O-containing precursor.

## Introduction

Carbon exhibits a remarkable propensity for forming nanomaterials with unusual physical and chemical properties arising from its ability to engage in different covalent bonding states^1–4^. Many of these next-generation nanomaterials, which include nanodiamonds, nanographite, amorphous nanocarbon, nano-onions, etc. are currently being studied for possible applications spanning quantum computing to bio-imaging^1^, and ongoing research suggests that high-pressure synthesis could lead to the discovery and possibly the tailored design of many more. Both laser-driven shock^5,6^ and detonation experiments^3,7,8^ can be used to drive carbon-rich materials to the 1000s of K and 10s of GPa conditions under which complex processes lead to the formation of 2–10-nm nanocarbons within 100s of nanoseconds. However, the precise chemical and physical phenomena governing nanocarbon formation under elevated pressure and temperature largely remain *terra incognita*, due in part to the formidable challenges associated with studying systems at such extreme states. Recent experiments on nanodiamond production from hydrocarbons subjected to conditions similar to those of planetary interiors^5^ offer some clues on possible carbon condensation mechanisms, but the landscape of systems and conditions under which intense compression could yield interesting nanomaterials is too vast to be explored using experiments alone.

Atomistic simulations can be leveraged to provide insight into the fundamental processes controlling formation of nanocarbon materials, and could also serve as a design tool to help guide experimental efforts. However, the associated temporal and spatial scales are inaccessible to highly-predictive first-principles-based approaches, e.g., Kohn–Sham density functional theory (DFT), while molecular mechanics models applicable to materials exposed to extreme temperatures and pressures are quite scarce. Recently, we proposed a new machine-learned interatomic model and modeling paradigm, the Chebyshev Interaction Model for Efficient Simulations (ChIMES)^9–15^, which is capable of retaining “quantum accuracy” while maintaining the computational efficiency of a molecular mechanics approach.

In this paper, we significantly advance the modeling effort initiated in ref. ^6^ by performing an in-depth investigation of carbon condensation (precipitation) in oxygen-deficient C/O mixtures at high pressures and temperatures. To this end, we leveraged the ChIMES framework to both substantially extend the simulations of ref. ^6^ and perform new simulations at different C/O ratios. C/O systems are often employed in practical applications and are excellent candidates for uncovering the essential features of this complex process due to their relative simplicity. For example, carbon monoxide (CO) has been used for carbon nanotube production under ambient to slightly elevated pressures, and explored as a precursor for other nanocarbon materials^16–18^, while shock compression of liquid CO to 10s of GPa has yielded convincing evidence of nanocarbon formation^6,19^. Furthermore, C/O systems serve as a logical stepping stone to the CNO (and CHNO) organic compounds traditionally employed for high-pressure nanodiamond synthesis (e.g., benzotrifuroxan, C_6_N_6_O_6_)^8^, or those recently shown to yield carbon nano-onions (e.g., bis(nitrofurazano)furazan, C_6_N_8_O_8_)^7^.

Carbon condensation in organic systems subject to high temperatures and pressures is clearly a non-equilibrium process akin to phase separation in mixtures quenched from a homogenous phase into a two-phase region^20,21^, yet this connection has only been partially explored^22–24^; notably, phase separation concepts remain very relevant for nanoparticle synthesis^25^. Chemical bonding adds a new dimension to the classical problem of segregation kinetics and can generate interesting and consequential features, e.g., by introducing length scales that determine system morphology^26^. Atomistic modeling could help elucidate such problems but the research remains in its infancy, with applications to carbon precipitation nearly nonexistent. Herein we employ the machine-learned ChIMES reactive interatomic potential framework to simulate carbon-rich C/O mixtures and address open questions surrounding formation of nanocarbon from highly compressed organic materials, e.g.:

- What is the role of chemistry during carbon condensation?
- How do carbon clusters evolve, both structurally and chemically, and what is their growth mechanism?
- How does overall system composition influence carbon cluster formation?

Our results provide an atomistically-resolved view of carbon clustering in a condensed phase reactive system and make possible for the first time a comprehensive analysis of this process, thereby opening new investigative avenues that could lead to in silico design of next-generation nanomaterials.

## Results

Unless otherwise stated, simulations were performed in the *NVT* ensemble at 6500 K and 2.5 g/cm^3^. These conditions approximately correspond to the Hugoniot state point achieved by a 7 km/s shock into cryogenic liquid CO, thereby mimicking the classic experiments of Nellis and coworkers^19^ that first suggested carbon precipitation from a shocked organic liquid. All simulations were run using the LAMMPS^27^ software suite, and interatomic interactions were evaluated using the ChIMES interatomic potential^12^ via the ChIMES calculator library (https://github.com/rk-lindsey/chimes_calculator). These models, which are machine-learned to Kohn–Sham density functional theory^28^ (DFT), are capable of yielding quantum accurate predictions for structure, dynamics, and speciation, provide several-orders-of-magnitude increases in efficiency over first-principles methods, and exhibit computational efficiency that scales linearly with system size. We note that additional simulation details are provided in the “Methods” section.

In this work, carbon condensates are defined as any set of carbon atoms containing at least 10 members, instantaneously separated by no more than *r*_CC_ = 1.9 Å from their nearest neighbor within a candidate cluster (with *r*_CC_ corresponding to the location of the first minimum of the C–C radial pair distribution function, RDF). For clusters containing both atom types, oxygen atoms within *r*_CO_ = 1.8 Å of a cluster carbon were also counted, with *r*_CO_ taken as the location of the first minimum of the corresponding RDF. While the choice of clustering criteria may have some effect on predictions, e.g., on cluster radii, we note that our overall analysis and conclusions remain unchanged.

**Fig. 1:**
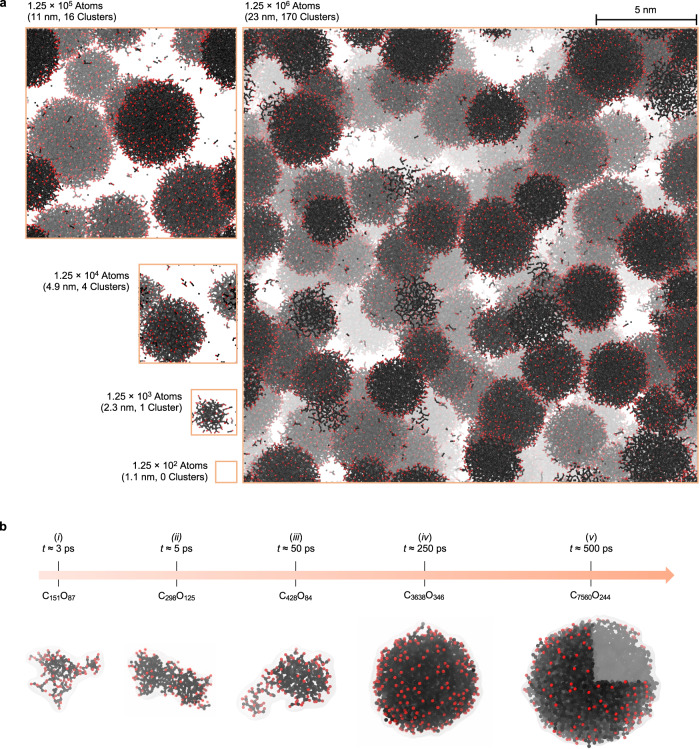
Cluster populations for different system sizes and typical cluster growth sequence. **a** Snapshots for systems containing ≈1.25 × 10^2^, 1.25 × 10^3^, 1.25 × 10^4^, 1.25 × 10^5^, and 1.25 × 10^6^ atoms at 0.25 ns. Images are to scale, and for clarity, only show atoms participating in cluster formation. **b** Representative cluster growth timeline (partially adapted from ref. ^6^, Fig. 4b). The light gray isosurface around each cluster is given to provide a sense of shape. A partial cross-section is provided for cluster *v* at time *t* ≈ 500 ps. In all images, red beads are oxygen atoms and black beads are carbon atoms; fog is used to convey depth.

Carbon condensation predictions from atomistic simulations are vulnerable to finite size effects^12,29^, since precipitation progresses through formation and evolution of a well-defined nano-sized carbon cluster population. As shown in Fig. 1a, simulations for 5 system sizes spanning 4 orders of magnitude (i.e., ≈1.25 × 10^2^ to 1.25 × 10^6^ atoms) were investigated to quantify the extent of such effects. We summarize salient findings here, noting that further discussion is available in the Supplementary Discussion. All systems containing more than ≈1.25 × 10^2^ atoms yielded at least one cluster, though clusters did not become morphologically and compositionally similar until system sizes reached at least 1.25 × 10^4^ atoms. This is notable within the context of recent studies of this system; ultra-fast laser-driven shock experiments, which probe a target system on similar scales (i.e., 1 μm and 10s to 100s of ps) have confirmed growth of nano-sized carbon condensates within 50 ps^6^. Conversely, recent DFT-MD simulations found only small covalent species of C_2*n*_O_*n*_ stoichiometry^29^, with *n* ≈ 21. The discrepancy between the DFT-MD result and those from the present simulations (and available experiments) ultimately stems from the high computational expense associated with DFT, which limits system scales to ${{{{{{{\mathcal{O}}}}}}}}$ 100 atoms and 10s of ps. Increasing system size in the current simulations has the primary effect of increasing the range of observed cluster dimensions. As expected, late-time cluster growth kinetics are significantly impacted by system size; nevertheless, the average cluster radii and qualitative behavior are consistent across all systems with ≥1.25 × 10^4^ atoms.

To determine how system composition impacts condensate formation, systems with eight C:O ratios were investigated in addition to the 1:1 C:O system described above, ranging from 95/5, to 25/75% CO/CO_2_. Condensates were found to form within 25 ps for all simulations with at least 60% CO. Data and additional discussion for these simulations is provided in the Supplementary Discussion and Supplementary Figs. 7 and 8. Briefly, we find that for condensate-producing compositions, increased oxygen concentration has the primary effect of slowing cluster formation kinetics. However, the formation mechanism, cluster properties, and qualitative growth kinetics are not significantly modified. Hence, we focus here on the 100% CO and 80/20% CO/CO_2_ systems, which we henceforth distinguish by their respective C:O ratios, i.e., 1:1 and 5:6. We note that, in contrast to the 1:1 case, cluster morphology, composition, and formation kinetics for the 5:6 C:O system were largely unaffected by system size, provided ≥1.25 × 10^4^ atoms were used. For the remainder of this work, simulation results for the 1:1 system will correspond to a single 500 ps simulation of 1.25 × 10^6^ atoms, whereas results for the 5:6 system are an average over 4 independent 250 ps simulations of 1.25 × 10^5^ atoms; when results are being compared between the 1:1 and 5:6 cases, data for both concentrations correspond to the latter simulation protocol, unless otherwise stated. We note that 5:6 C:O simulations were run at 6500 K and 2.46 g/cm^3^, yielding the same initial pressure as the 1:1 C:O simulation at 6500 K and 2.5 g/cm^3^.

### Cluster evolution

Figure 1b provides snapshots of representative carbon clusters during a typical growth progression, suggesting a few notable features of their evolution. At the early time (Fig. 1b *i*), small extended polymeric fragments are observed, typically showing C_2*n*_O_*n*_ stoichiometry, consistent with previous small-scale DFT studies^29^. As time passes (Fig. 1b *ii* and 1b *iii*), the clusters become more compact, begin to densify and expel interior oxygen, leading to the formation of large spherical liquid carbon droplets, with oxygen atoms located primarily on their surface (Fig. 1b *iv* and 1b *v*). Final snapshots for cluster-containing systems (i.e., Fig. 1a), suggest that smaller clusters are morphologically distinct from their larger counterparts, and increasing system size leads to a broader cluster size distribution.

**Fig. 2:**
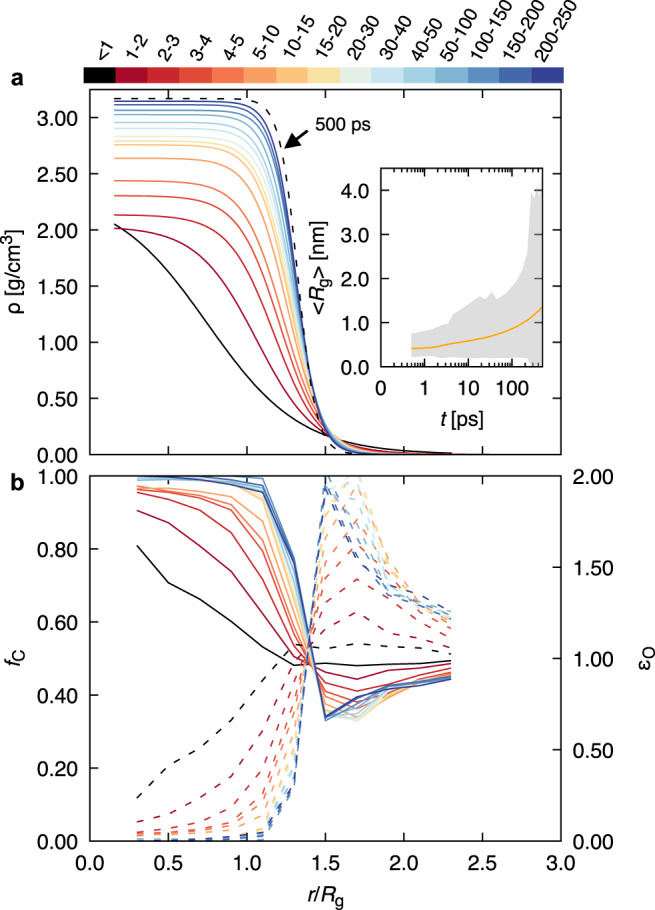
Average cluster density and composition profiles. **a** Reduced radial density profiles *ρ*(*r*/*R*_g_), and time-resolved average radius of gyration (inset), 〈*R*_g_〉, for the 1:1 C:O system. Plotted radii, 〈*R*〉, and density profiles correspond to hyperbolic tangent fits to each histogrammed *ρ*(*r*/*R*_g_). The black dashed curve gives the profile at 500 ps and the shaded region in the 〈*R*_g_〉 inset figure gives the maximum and minimum observed *R*_g_ at each time block, while the orange line gives the average value. **b** Radial atomic fraction of carbon (*f*_C_, solid lines) and oxygen enrichment (*ϵ*_O_ = *f*_O_/*f*_C_, dashed lines) emanating from cluster centroids. The color bars provide the corresponding time range for each line, in ps, unless otherwise specified.

A quantitative picture of cluster evolution is provided in Fig. 2, which shows reduced average radial density profiles, *ρ*(*r*/*R*_g_), for the 1:1 system cluster population, along with time-resolved average cluster radii of gyration *R*_g_ (i.e., the average root mean squared distance between cluster carbon atoms and cluster carbon center of mass). Note that plotted radii and density profiles correspond to hyperbolic tangent fits to each histogrammed *ρ*(*r*/*R*_g_) of the form: $\rho =0.5{\rho }_{0}\big\{1-\tanh \big[{{w}_{0}}^{-1}\big(r/{R}_{{{{{{{{\rm{g}}}}}}}}}-{d}_{0}\big)\big]\big\}$, with *w*_0_ = *w*/*R*_g_ and *d*_0_ = *d*/*R*_g_, where *ρ*_0_ is the density in the center of the cluster, *w* is the interfacial width, and *d* is the distance from the cluster centroid to the interface. (We note that the interface location, *d*, is a suitable measure for the cluster size, but it is numerically more difficult to determine precisely for individual clusters than *R*_g_, which we employ below).

For clarity, the present analysis considers only carbon atoms within a cluster; since oxygen is located almost exclusively at the exterior in mature clusters, this choice has minimal influence on late-time results. These profiles indicate a transition from extended, low-density clusters at early time to clusters with an interior density of ~3 g/cm^3^. At the same time, the cluster interface steepens considerably, with the interfacial width, *w*, decreasing from ≃2.50 to a stable value of ≃1.25 Å over 50 ps. Early time density profile curves correspond to clusters such as those in Fig. 1*i*, which exhibit approximately C_2*n*_O_*n*_ stoichiometry and appear to be of covalent nature (note that our models do not explicitly treat bonding as is common in molecular mechanics formalisms), whereas late clusters (Fig. 1*iv*) are best described as liquid carbon droplets. As shown in Fig. 2a (inset) the average cluster radius *R*_g_ increases with time, with the largest clusters reaching sizes of a few nanometers. Additional radial density profiles and average radii of gyration for different C:O ratios are available in Supplementary Figs. 6 and 8. In general, we find that cluster structural evolution is mostly uncoupled from system composition.

Cluster chemical composition evolution is a crucial aspect of carbon precipitation and growth kinetics. Time-resolved, radial atomic fractions emanating from cluster centroids are given in Fig. 2b. Note that these curves are obtained by considering all atoms in the system within *r*/*R*_g_ of a given cluster centroid. We find that at early times cluster interiors (i.e., *r*/*R*_g_ < 1) have a sizable oxygen content, with values as large as 20%. As time progresses, oxygen migrates to the exterior, resulting in essentially pure liquid carbon droplets, surrounded by carbon depletion and oxygen enrichment layers with approximate CO_2_ stoichiometry. The amount of oxygen within the growing clusters appears to decay exponentially in time, with a relaxation time constant of ≃5 ps (see Supplementary Fig. 3). A similar analysis of the 5:6 C:O system (see Supplementary Fig. 6) shows that the overall C:O ratio has little qualitative effect on the cluster evolution, indicating that under these conditions the densification and oxygen elimination processes are not very sensitive to system stoichiometry. As the average cluster composition stabilizes, so does the makeup of the reactive fluid that the clusters are immersed in; time-resolved molecular speciation, shows that this is chiefly comprised of CO_2_, CO, and free oxygen atoms at late times (see Supplementary Fig. 1).

**Fig. 3:**
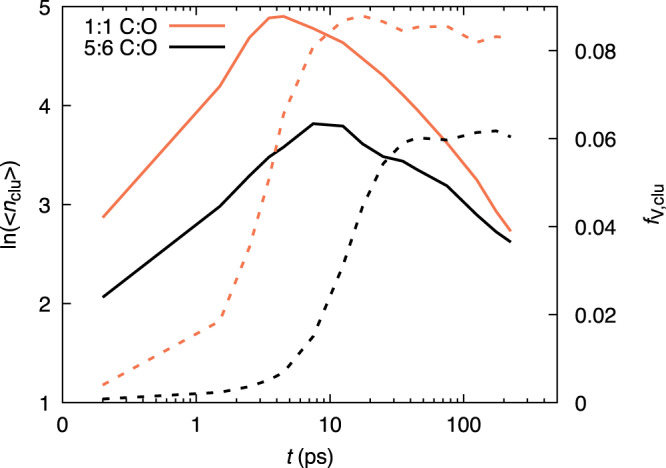
Total number and volume fraction of clusters. Time (*t*) resolved natural log of the average number of clusters, ${{{{{{{\rm{\ln }}}}}}}}\left(\langle {n}_{{{{{{{{\rm{clu}}}}}}}}}\rangle \right)$ (solid lines), and average overall cluster-volume fraction, 〈*f*_v,clu_〉 (dashed lines) in the 1:1 and 5:6 CO systems, as indicated by line color.

As shown in Fig. 3, the total cluster-volume fraction, 〈*f*_v,clu_〉, calculated from individual cluster radii of gyration, exhibits behavior characteristic of precipitation from a supersaturated solution, with an initial delay followed by step-like growth and rapid leveling off to a value dependent on the system composition. The delay is slightly longer in the 5:6 system relative to the 1:1 case, likely due to the lower initial carbon supersaturation, while the final condensed carbon volume fraction is smaller. It is worth noting that the observed supersaturation dependence of the delay is consistent with classical nucleation effects in mixtures^30^, which determine the persistence of an initial metastable, single fluid phase in a two-phase region.

As already noted in ref. ^6^, the volume fraction for the 1:1 system at ≃50 ps (≃8%) agrees with experimental results for CO compressed on timescales of 100s of ps to thermodynamic conditions similar to these simulations, and with chemical equilibrium calculations that predict the end state of the system^6^. Figure 3 indicates that the transition to near-constant total cluster-volume fraction coincides with a maximum in the number of clusters present in the system for both systems discussed here (1:1 and 5:6 C:O ratios), suggesting that the later time evolution is dominated by coarsening kinetics.

### Growth kinetics

**Fig. 4:**
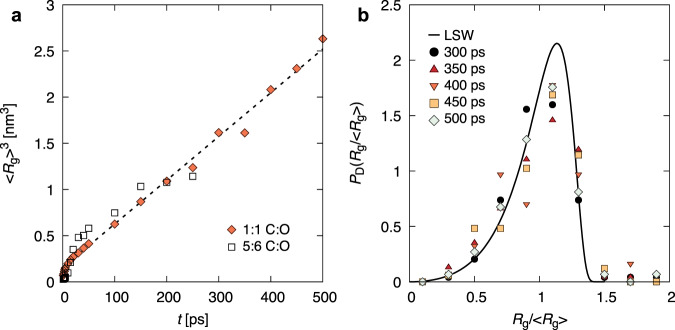
Cluster growth and size distribution. **a** Cubed average radius of gyration, ${\langle {R}_{{{{{{{{\rm{g}}}}}}}}}\rangle }^{3}$, as a function of time, *t*, for the 1:1 and 5:6 C:O systems (data for the 1:1 system with *t* ≤ 250 ps is adapted from ref. ^6^, Fig. 4b). The dashed line provides a linear fit to *t* = 5 to 500 ps and is only intended to serve as a guide to the eye. **b** Cluster size distributions, *P*_D_, in terms of reduced cluster radii of gyration, *R*_g_/〈*R*_g_〉, for *t* = 300 to 500 ps in the 1.25 × 10^6^ atom 1:1 C:O system. The cluster size distribution predicted by Lifshitz, Slyozov, and Wagner (LSW) theory is given by the solid black line.

We quantify cluster growth kinetics through analysis of the average cluster radius 〈*R*_g_〉 as a function of time. Figure 4a shows that at late times both systems (i.e., 1:1 and 5:6 C:O) exhibit linear growth in the average cluster volume, ${\langle {R}_{{{{{{{{\rm{g}}}}}}}}}\rangle }^{3}$ vs *t*, i.e., $\langle {R}_{{{{{{{{\rm{g}}}}}}}}}\rangle \propto {t}^{\frac{1}{3}}$. This behavior is consistent with coarsening via Ostwald ripening (i.e., evaporation–condensation or dissolution–redeposition mechanism), described by Lifshitz, Slyozov, and Wagner (LSW)^31–33^, in which diffusional mass transfer occurs between the clusters, with clusters smaller than the average size shrinking and the larger ones growing. The present results thus establish the relevance of LSW phenomenology for carbon condensation in a high-pressure–high-temperature chemically reactive environment. We note that an important feature of the LSW mechanism is that the average cluster size is the current critical nucleus size for the system. This interpretation is likely valid for the present case as well; the idea was employed in ref. ^24^. Hydrodynamic effects are known to determine fluid phase separations at large, ≈0.5 volume fractions of the minority phase (here carbon)^34^; for intermediate values, direct cluster coagulation (Brownian aggregation) is expected to yield volume fraction-dependent growth^34,35^, which is believed to be dominant in detonation-driven carbon condensation on long (${{{{{{{\mathcal{O}}}}}}}}\,100s$ ns) timescales^22,23,36^. Such scales are beyond the practical reach of our atomistic simulations. Nevertheless, we note that the LSW mechanism identified here plays a crucial role in cluster coagulation by inducing composition correlations between neighboring clusters which drive their direct aggregation^37^; a possible example is in ref. ^36^.

**Fig. 5:**
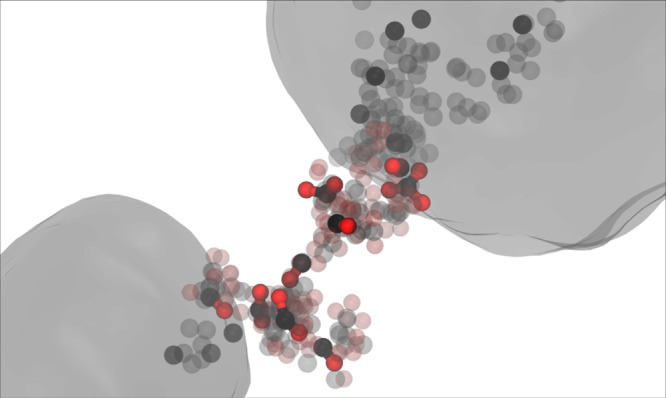
Reactive transport trajectory of a single carbon atom (black) from a small (lower left) to a relatively larger cluster (upper right). Large transparent blobs provide the approximate cluster carbon isosurface in a single frame, and oxygen atoms bonded to the carbon atom during transport are colored in red. For visual clarity, only a handful of frames showing the tracked carbon atom (and oxygen atoms bonded to it) have been rendered opaquely. A movie of this process is available as Supplementary Information.

Coarsening in phase separating systems is known to exhibit dynamical scaling, characterized by a time-invariant cluster population when scaled by the average cluster size^20,21^. We find that the carbon cluster size distribution in the current simulations is largely self-similar at late times, and compares well with the original LSW distribution^31^ (see Fig. 4b); the predominance of the dynamical scaling regime on sub-nanosecond timescales has important implications for modeling the carbon condensation process and its practical applications (e.g., nanoparticle synthesis or detonation). Note that differences are likely due to volume fraction effects^33^. This further suggests that cluster growth through diffusive flux is indeed the leading coarsening mechanism. However, we note that in the present system the mass transfer process is more complex than in classical fluid or solid solutions, in which minority phase atoms evaporate from smaller clusters, diffuse through the surrounding solvent matrix and condense on larger clusters. In particular, the carbon clusters are immersed in a reactive fluid primarily composed of CO_2_, CO, and O with virtually no free C atoms. Given the properties of the oxygen-rich cluster surface layer (see Fig. 2) and the makeup of the reactive fluid, carbon transfer between the clusters appears to principally involve the reactions C_*n*_ + CO_2_ ↔ C_*n*−1_ + 2CO and C_*n*_ + O ↔ C_*n*−1_ + CO, proceeding predominantly forward for small clusters and backward for larger ones, with CO and CO_2_ being the main carbon carriers between the clusters. We illustrate this atomic-level hitchhiking process in Fig. 5, which shows the chemical bonding environments of a single carbon atom as it travels from one cluster to another. As can be seen in the figure (and corresponding Supplementary Movie), the carbon atom shuttling between clusters reacts throughout its journey, associating with different oxygen atoms along the way to form CO and CO_2_ molecules. This chemical picture likely becomes much more complex for organic systems that also contain nitrogen and/or hydrogen^15^. Subsequent system quenching to lower pressures and temperatures (e.g., occurring in the wake of a shockwave) will have significant impact on the carbon condensation process, both through the phase transformation of the carbon nanodroplets to other carbon nanoallotropes, e.g., nanodiamond, and possible inhibition of chemical reactions between the clusters and the surrounding reactive fluid. These effects will play an important role in determining the properties of clusters that are ultimately recovered from experiments. Simulation studies of this quenching process will be the subject of future investigations.

## Discussion

We have presented atomistic simulations of chemistry-coupled carbon condensation and an in-depth analysis addressing long-standing questions related to high-pressure nanocarbon synthesis in organic systems. We find that this process can be suppressed by small system sizes, e.g., hundreds of atoms typically accessible to DFT-based methods, and that much larger simulations are needed to capture its physicochemical features. Our simulations have yielded a comprehensive picture of carbon cluster evolution in carbon-rich C:O systems at extreme conditions, which is surprisingly similar to canonical phase separation in fluid mixtures, but also exhibits unique features typical of reactive systems. At early times extended, polymeric seed clusters of approximate C_2*n*_O_*n*_ composition form rapidly as excess carbon precipitates out of the supersaturated solution. At the next stage, these clusters densify by expelling interior oxygen and become spherical, ultimately exhibiting purely molten-carbon interiors and oxygen-decorated exteriors. Clusters occupy ≃8% of system volume in the 1:1 C:O system, and possess interior densities of ≃3 g/cm^3^, typical of liquid carbon. We also find that changing composition (i.e., by increasing oxygen concentration) has the primary effect of hindering formation kinetics but does not otherwise appear to significantly impact cluster composition, governing formation chemistry, or growth kinetics. Their growth on hundreds of picoseconds timescales occurs mainly through Ostwald ripening and generates cluster populations that obey dynamical scaling. However, in contrast to the classical picture prevailing in simple solid or fluid mixtures, inter-cluster carbon diffusive transport is chemistry-mediated and may perhaps be described as atomic hitchhiking via chemical bonding. These features are likely to play an important role in predicting and possibly controlling the condensation process and properties of resulting nanocarbon particles. The present simulation approach can be extended to CNO^15^ and CHNO systems, and could eventually be leveraged as an in silico material design tool, substantially lowering experimental barriers associated with creating and refining carbon nanoallotrope synthesis protocols.

## Methods

Simulations were initialized as a fluid of randomly packed CO and CO_2_ molecules at a ratio yielding the target C/O composition, and allowed to relax for 10 ps at ≃100 K to reduce unfavorable close contact due to packing; velocities were then rescaled yielding a system temperature of 6500 K. We note that system initialization protocols have a negligible influence on simulation predictions for this work due to the rapid dynamics occurring under such elevated temperatures. For example, results from exploratory simulations initialized as a system of randomly packed C and O atoms and those from exploratory simulations initialized from equilibrated DFT-MD simulations at 6500 K and 2.5 g/cm^3^ were statistically indistinguishable. All simulations were run using the LAMMPS^27^ software suite, and interatomic interactions were evaluated using the ChIMES interatomic potential^12^. These models, which are machine-learned to Kohn–Sham density functional theory^28^ (DFT), are capable of yielding quantum accurate predictions for structure, dynamics, and speciation, provide several-orders-of-magnitude increases in efficiency over first-principles methods, and exhibit computational efficiency that scales linearly with system size. A detailed description and validation of the ChIMES model for this system are available in ref. ^12^. We note that the ChIMES model utilized herein, a library for computing ChIMES interactions, and source code necessary for its use within LAMMPS, are available at https://github.com/rk-lindsey/chimes_calculator.

## Supplementary information

Supplementary Information

Description of Additional Supplementary Files

Supplementary Movie 1

## Data Availability

The simulation datasets generated and/or analyzed in the current study are available upon reasonable request, due to the large associated data volumes. MD simulations were performed using the LAMMPS software package, available at https://github.com/lammps/lammps. The library used for computing ChIMES interactions and parameter set employed in this work are available at https://github.com/rk-lindsey/chimes_calculator.
