# Supplementary material for: Chemistry-mediated Ostwald ripening in carbon-rich C/O systems at extreme conditions
Source: Nat Commun. 2022 Mar 17;13:1424. doi: 10.1038/s41467-022-29024-x (PMC8931168; doi:10.1038/s41467-022-29024-x)
Supplement: Supplementary file 1 — Supplementary Information [file 41467_2022_29024_MOESM1_ESM.pdf]

Supplementary Information: Chemistry-Mediated Ostwald Ripening in Carbon-Rich C/O Systems at Extreme Conditions

R. K. Lindsey et al.

Supplementary Discussion

Here we provide an overview of the chemical evolution occurring in the reactive fluid during the appearance and growth of the carbon clusters. As shown in panel a of Supplementary Figure 1, the fluid surrounding the clusters is comprised of a variety of small species, though only CO, CO_2, and O are present in notable quantities. Figure 1 panel b indicates that these small species account for approximately 20% of all carbon in the system, at any given time. The remaining carbon is distributed between clusters, i.e. any set of carbon atoms containing at least 10 members separated by no more than $r_{CC} = 1.9$ Å and "poly" material, i.e. species that are neither nominal clusters nor the small molecules mentioned above. We note that at early times this "poly" material encompasses the polymeric fragments which are precursors to the first carbon clusters, while at late times it contains the remnants of clusters that have been consumed via Ostwald ripening.

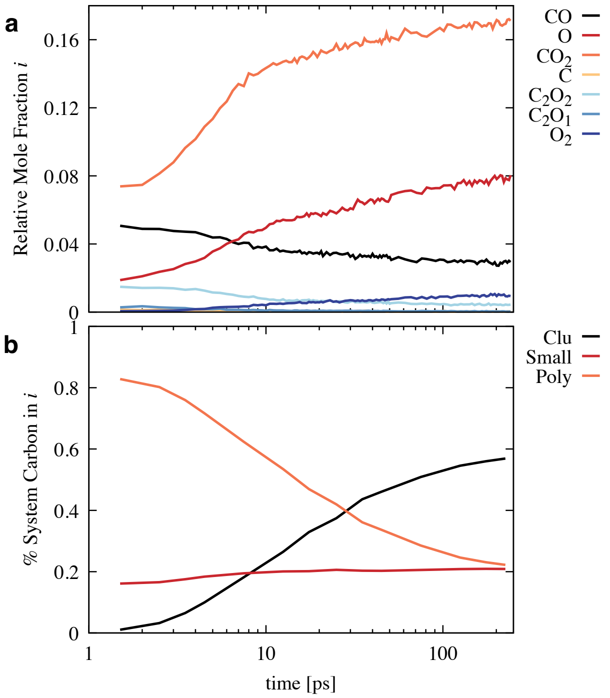

Supplementary Figure. 1: Panel a: Instantaneous relative mole fractions of small species, i, found in the 1.25×10^6 atom 1:1 C:O system. Panel b: Fraction of carbon in the 1.25×10^6 atom 1:1 C:O system contained in small species i, clusters, and "poly" species of intermediate size (i.e. $2 < n_C < 10$, where n_C is the number of constituent carbon atoms).

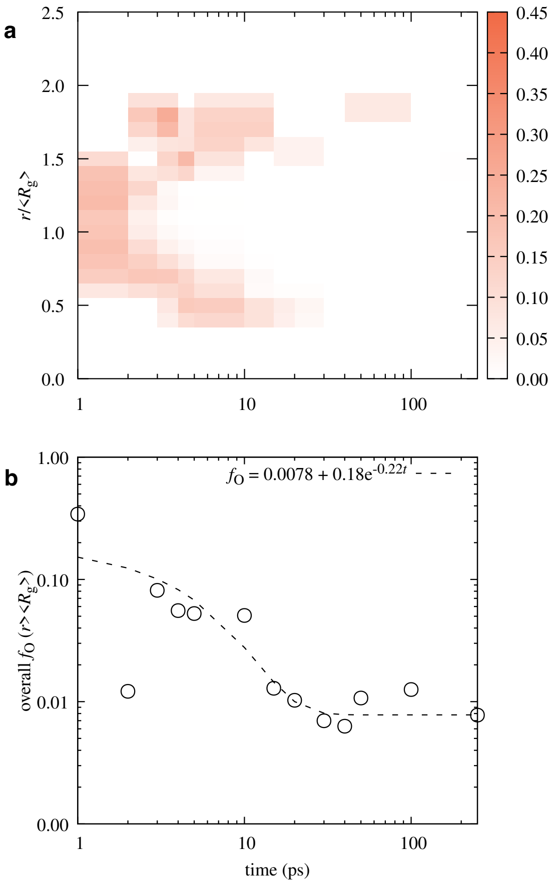

Supplementary Figure. 2: Panel a: Time resolved location ($r/\langle R_g \rangle$) and atomic fraction of oxygen (color) averaged over all clusters. Panel b: Oxygen elimination kinetics for growing clusters in terms of overall fraction of atomic oxygen, f_O for $r > \langle R_g \rangle$.

As noted in the main manuscript, an important part of the cluster evolution is the elimination of interior oxygen; Supplementary Figure 2 provides an overview of this carbon cluster purification kinetics. Panel a shows a spatially-resolved view of the oxygen content averaged over all the clusters, indicating that initially, oxygen is fairly abundant and largely uniformly distributed. As time progresses, oxygen is found to migrate to cluster exteriors (i.e. larger $r/\langle R_G \rangle$ values). The apparent concomitant oxygen localization at cluster interiors shown in panel a of Supplementary Figure 2 before $\simeq$ 10 ps likely arises from small clusters merging via direct contact (aggregation).

We quantified the elimination kinetics by plotting the amount of oxygen in all clusters larger than the average $r/\langle R_G \rangle$ (i.e. clusters growing via Ostwald ripening), divided by the total number of atoms in these clusters, as a function of time (Supplemen-

tary Figure 2, panel b). The resulting data are well described by a simple exponential relaxation model ($x = a_0 + a_1 e^{-a_2 t}$, where $a_0 = 0.0078$, $a_1 = 0.18$, and $a_2 = 0.22$); this indicates that the growing clusters likely contain a small fraction of impurity oxygen (i.e. <1%), suggesting non-zero solubility of oxygen in liquid carbon at these conditions.

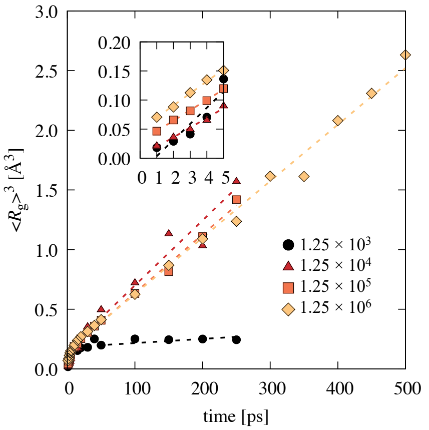

Supplementary Figure. 3: Cubed average radius of gyration, $\langle R_\text{g} \rangle^3$, as a function of time for systems containing 1.25×10^3, 1.25×10^4, 1.25×10^5, and 1.25×10^6 atoms. Dashed lines provide linear fits to either $t = 0$ to 5 ps or $t = 5$ to 250 ps and are only intended to serve as a guide to the eye.

Supplementary Figure 3, shows that each system with more than $\approx 1.25 \times 10^3$ atoms follows the classical cluster growth trend discussed in the main text (i.e. linear increase in $\langle R_\text{g} \rangle^3$ with time). In the three larger systems, we observe two distinct regimes, distinguished by the inflection point at $t \approx 5$ ps separating initial appearance and growth stages, whereas for the smallest cluster-containing system ($\approx 1.25 \times 10^3$ atoms), three regimes emerge. The final flat linear portion of the latter data set is attributed to system size effects, where formation of a single cluster and ensuing growth saturation is observed. Linear fits to the data indicate that growth kinetics are consistent for the larger systems.

We note that the classical growth kinetics observed for the present system enables prediction of $\langle R_\text{g} \rangle$ as a function of time, and comparisons with carbon recovery experiments. For example, our recent UF laser-drive shock experiments on CO [1] yielded amorphous carbon clusters of $r = 2.5$–15 nm, [1] suggesting onset quenching times of $\approx$ 1–2 ns, in agreement with the experimental time scales.

We provide the average cluster reduced radial density profiles, $\rho(r/R_\text{g})$ and radii of gyration, R_g in Supplementary Figure 4 panels a through d, for each system with at least 1.25×10^3 atoms. We note that, for clarity, the present analysis considers only carbon atoms within a cluster; since oxygen is located exclusively at the exterior in mature clusters, this assumption has minimal influence on later-time results. In

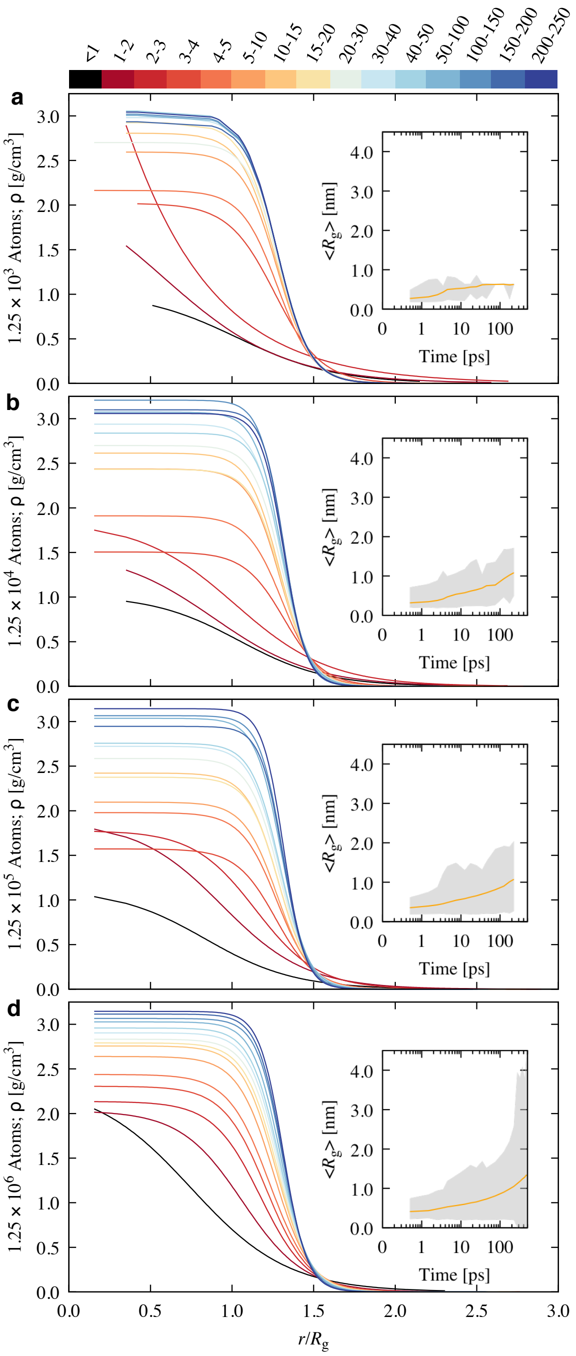

Supplementary Figure. 4: Reduced radial density profiles, $\rho(r/R_\text{g})$, and time-resolved average radius of gyration (inset), $\langle R_\text{g} \rangle$, for systems containing 1.25×10^3, 1.25×10^4, 1.25×10^5, and 1.25×10^6 atoms. The color bar at the top of the figure provides the time range (in ps) utilized in constructing each density profile. The shaded region in the $\langle R_\text{g} \rangle$ inset figures gives the maximum and minimum observed R_g at each time block, while the orange line gives the average value.

order to generate a given *reduced* radial density profile, a radial density profile was computed for each relevant cluster. A $\rho(r/R_g)$ histogram was then constructed by binning each density profile according to r/R_g. For clarity, the results presented herein correspond to hyperbolic tangent fits (see main text) to each histogrammed $\rho(r/R_g)$.

For all system sizes, average cluster profiles - Supplementary Figure 4 - transition from extended, low-density curves at early time to curves indicating an interior density of approximately 3 g/cm^3, with a steep cluster interface. We also find that in all but the 1.25×10^3 atom system, which forms a single cluster, average cluster radius of gyration, $\langle R_g \rangle$ and minimum radius of gyration, $R_{g,\mathrm{min}}$ values are consistent, with the former value increasing from approximately 0.4 to 1.0 nm in the first 0.25 ps; $R_{g,\mathrm{max}}$ values increase with system size.

Here we investigate the effects of system size on emergent system kinetics through time-resolved analysis of i. percentage of system carbon participating in cluster formation, $\langle \%C_{\mathrm{sys,clu}} \rangle$ (Supplementary Figure 5 panel a), ii. total cluster volume fraction, $\langle f_{\mathrm{v,clu}} \rangle$ (Supplementary Figure 5 panel b), and iii. total number of clusters present in the system, $\langle n_{\mathrm{clu}} \rangle$ (Supplementary Figure 5 panel c). Note that $\langle f_{\mathrm{v,clu}} \rangle$ is computed based on cluster R_g values. The results indicate that the evolution trends for each quantity are similar for different system sizes and consistent with Ostwald ripening evolution, and that the number of clusters is most severely affected by finite size effects. The late time values for the percentage of participating carbon and total volume fraction of clusters are in agreement for all systems, and appear to converge as the system size is increased.

Atomic composition plays a key role in determining carbon clustering kinetics. For example, energetic materials with different oxygen balance generally exhibit detonation properties differences that can be attributed in part to distinct carbon condensation effects.[2] As noted in the main text, introducing a third (and possibly forth) atom type in our current modeling paradigm should make such simulation studies possible. As a stepping stone to these future efforts we investigate the effects of decreasing the C:O ratio (from 1:1) on the carbon precipitation kinetics. We note that astrophysical studies have suggested that the C:O ratio of both rocky planets and their stars (i.e. under extreme temperature and pressure) is critical for determining overall mineralogy.[3]

The present studies were conducted for systems of $\approx 1.25 \times 10^4$ atoms containing 5, 10, 15, 30, 40, 50, and 75% CO_2, and the balance CO. (CO_2% and CO as utilized here strictly refer to initial concentrations.) Each of these simulations were conducted at 6500 K and the density determined to yield the same initial pressure as the 0% CO_2 simulations discussed in the main text. Although the system size is relatively small, finite size effects are generally less significant in systems with greater oxygen concentration (less excess carbon). We note that no cluster formation was observed over 0.25 ns for systems with $CO_2 \geq 50\%$, suggesting that at these thermodynamic conditions they are in a single phase region.

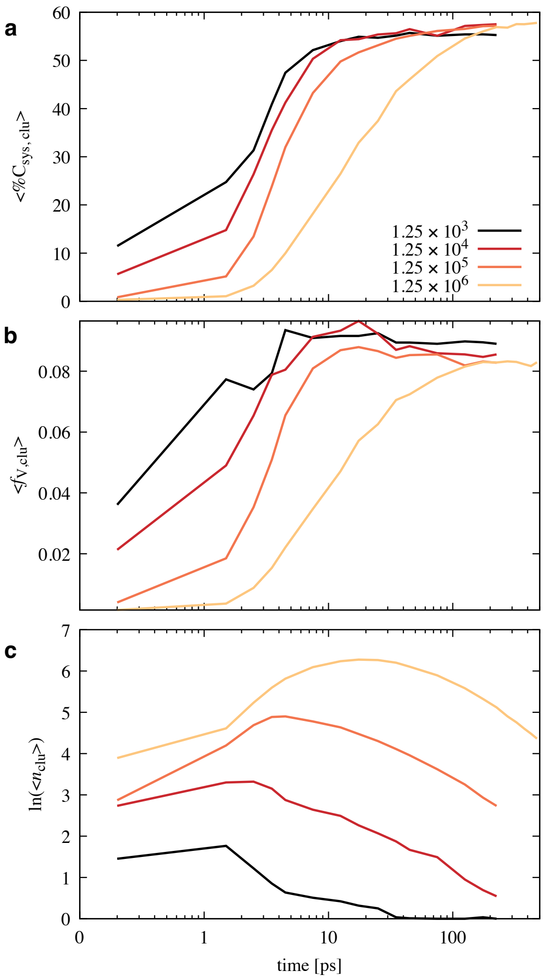

Supplementary Figure. 5: From top to bottom: average percentage of system carbon participating in cluster formation, $\langle \%C_{\mathrm{sys,clu}} \rangle$, average cluster volume fraction, $\langle f_{\mathrm{v,clu}} \rangle$, and natural log of the average number of clusters, $\ln\left(\langle n_{\mathrm{clu}} \rangle\right)$ for systems containing 1.25×10^3, 1.25×10^4, 1.25×10^5, and 1.25×10^6 atoms.

Supplementary Figure 6 shows radial composition in the 5:6 C:O system. Cluster chemical evolution proceeds in close similarity with the 1:1 C:O case described in the main text, with early time clusters exhibiting approximately 20% oxygen-containing interiors, and purifying to nearly neat carbon interiors at late time. The 5:6 system also exhibits formation

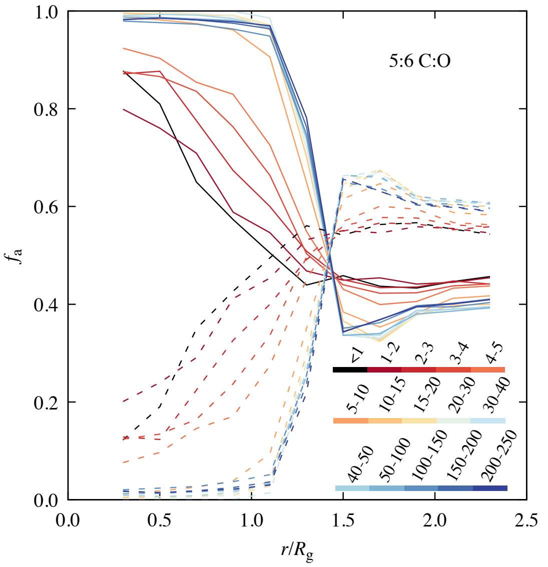

Supplementary Figure. 6: Radial (r/R_g) atomic fraction, f_a, of carbon (solid lines) and oxygen (dashed lines) emanating from cluster centroids, in the 5:6 C:O system. The color bars provide the corresponding time range for each line, in ps.

of an oxygen enrichment surface layer, which narrows (relative to the cluster radius) as the cluster matures. The primary difference compared with the 1:1 system is the C/O fractional composition at large r/R_g.

Supplementary Figure 7 shows that maximum values of $\langle\%C_{sys,clu}\rangle$ (panel a) and $\langle f_{v,clu}\rangle$ (panel b) are found to decrease as initial CO_2 content is increased from 0 to 40% (C:O ratio decreased from 1:1 to 1:1.4), leveling off at approximately 57% (9%) and 45%(5%), respectively. Systems containing less carbon were found to produce fewer clusters, and to undergo slower kinetics (see Supplementary Figure 7 panel c and Supplementary Figure 8 panels a through c) during the first $\approx$ 50 ps, likely due to nucleation effects. At later times, minimal differences in the evolution of average, minimum, and maximum R_G are observed, as expected for Ostwald ripening kinetics, with the exception of the $CO_2 = 40$ % case, where a single cluster is formed.

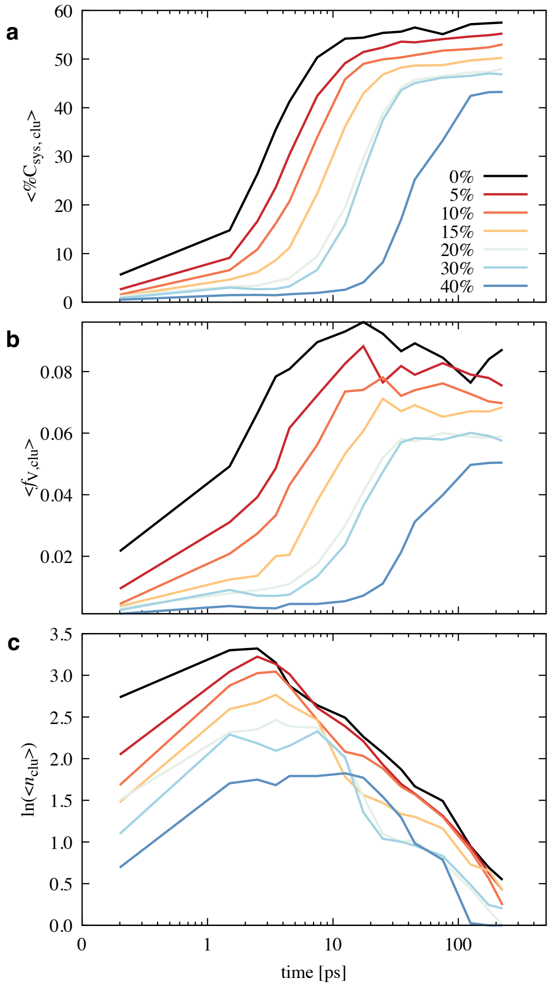

Supplementary Figure. 7: From top to bottom: average percentage of system carbon participating in cluster formation, $\langle\%C_{sys,clu}\rangle$, average cluster volume fraction, $\langle f_{v,clu}\rangle$, and natural log of the average number of clusters, $\ln(\langle n_{clu}\rangle)$ for systems containing $\approx 1.25\times10^4$ atoms, with 0 to 40% of the initial composition CO_2 (as indicated by line color) and the balance, CO.

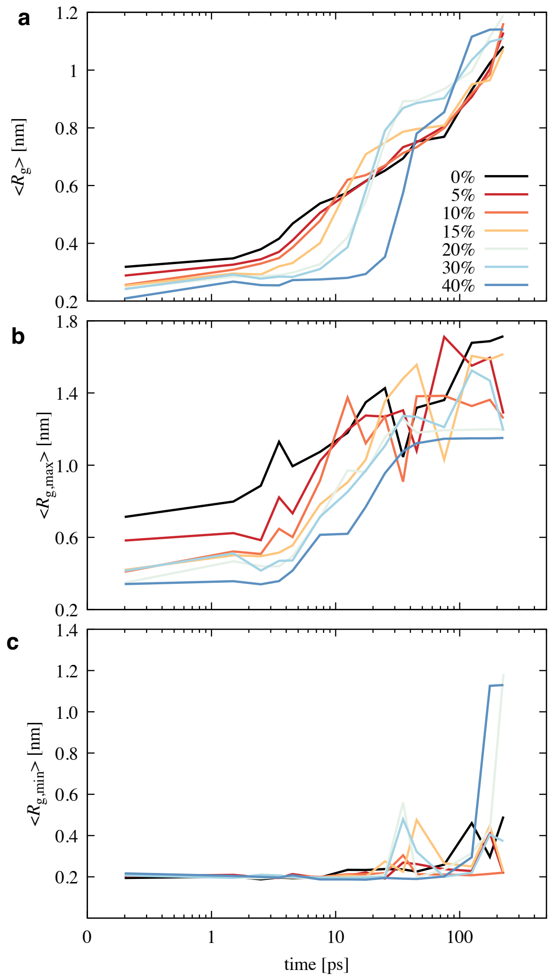

Supplementary Figure. 8: From top to bottom: Average ($\langle R_g \rangle$), average maximum $\langle R_{g,max} \rangle$), and average minimum $\langle R_{g,min} \rangle$) cluster radius of gyration for systems of varied CO:CO_2 ratio. Line color indicates CO_2%.

Supplementary References

(1) Armstrong, M.; Lindsey, R.; Goldman, N.; Nielsen, M.; Stavrou, E.; Fried, L.; Zaug, J.; Bastea, S. Ultrafast shock synthesis of nanocarbon from a liquid precursor. *Nature Communications* **2020**, *11*, 353.

(2) Bastea, S. Chemical equilibrium and carbon kinetics in explosives. *Proceedings of the 15th International Detonation Symposium* **2014**, 896.

(3) Mena, E. D.; Israelian, G.; Hernández, J. G.; Bond, J. C.; Santos, N. C.; Udry, S.; Mayor, M. Chemical clues on the formation of planetary systems: C/O versus Mg/Si for HARPS GTO sample. *The Astrophysical Journal* **2010**, *725*, 2349.
