# Supplementary material for: Chemistry-mediated Ostwald ripening in carbon-rich C/O systems at extreme conditions
Source: Nat Commun. 2022 Mar 17;13:1424. doi: 10.1038/s41467-022-29024-x (PMC8931168; doi:10.1038/s41467-022-29024-x)
Supplement: Supplementary file 2 — Description of Additional Supplementary Files [file 41467_2022_29024_MOESM2_ESM.docx]

**Description of Additional Supplementary Files**

**File Name: Supplementary Movie 1
Description:** Reactive transport of a single carbon atom (small black bead) between two clusters (opaque blobs, bottom left and top right). Oxygen atoms (small red beads) instantaneously bonded to the carbon atom are also shown. Note that, when the carbon is close to the cluster surface, bonded oxygen atoms are part of the cluster's diffuse outer oxygen layer.
