# Supplementary figures and images for: Chemistry-mediated Ostwald ripening in carbon-rich C/O systems at extreme conditions
Source: Nat Commun. 2022 Mar 17;13:1424. doi: 10.1038/s41467-022-29024-x (PMC8931168; doi:10.1038/s41467-022-29024-x)

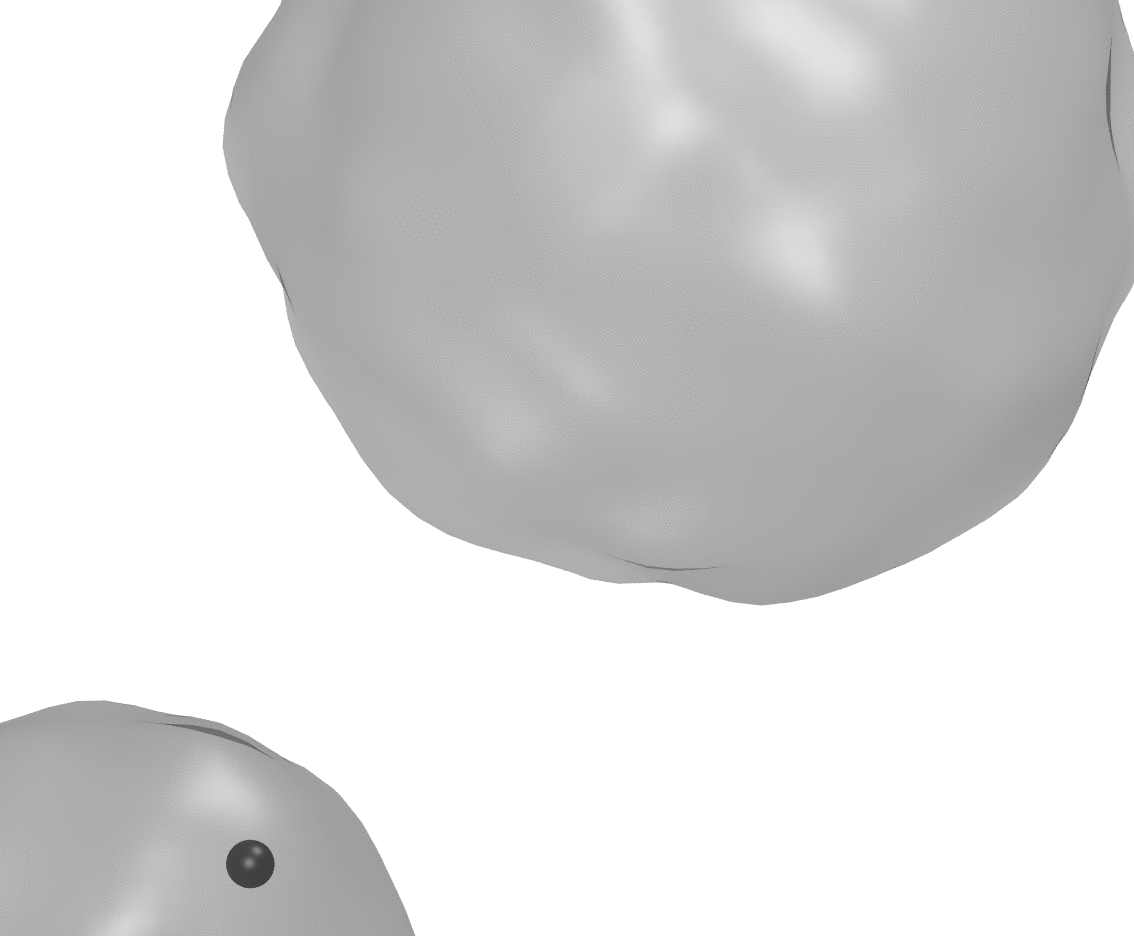

Supplement: Supplementary file 3 — Supplementary Movie 1 [file 41467_2022_29024_MOESM3_ESM.gif]
